# Challenges in maintaining medicine quality while aiming for universal health coverage: a qualitative analysis from Indonesia

**DOI:** 10.1136/bmjgh-2020-003663

**Published:** 2021-05-28

**Authors:** Amalia Hasnida, Maarten Olivier Kok, Elizabeth Pisani

**Affiliations:** 1 Health Care Governance, Erasmus School of Health Policy and Management, Erasmus University Rotterdam, Rotterdam, Zuid-Holland, Netherlands; 2 Migunani Research Institute, Yogyakarta, Indonesia; 3 Health Sciences, Vrije Universiteit Amsterdam, Amsterdam, Netherlands; 4 Policy Institute, King's College London, London, London, UK

**Keywords:** public health, health policy, health systems, health insurance, qualitative study

## Abstract

**Introduction:**

Indonesia, the world’s fourth most populous nation, is close to achieving universal health coverage (UHC). A widely-publicised falsified vaccine case in 2016, coupled with a significant financial deficit in the national insurance system, has contributed to concern that the rapid scale-up of UHC might undermine medicine quality. We investigated the political and economic factors that drive production and trade of poor-quality medicines in Indonesia.

**Methods:**

We reviewed academic publications, government regulations, technical agency documents and news reports to develop a semi-structured questionnaire. We interviewed healthcare providers, policy-makers, medicine regulators, pharmaceutical manufacturers, patients and academics (n=31). We included those with in-depth knowledge about the falsified vaccine case or the pharmaceutical business, medicine regulation, prescribing practice and the implementation of UHC. We coded data using NVivo software and analysed by constant comparative method.

**Results:**

The scale-up of UHC has cut revenues for physicians and pharmaceutical manufacturers. In the vaccine case, free, quality-assured vaccines were available but some physicians, seeking extra revenue, promoted expensive alternatives. Taking advantage of poor governance in private hospitals, they purchased cut-price ‘vaccines’ from freelance salespeople.

A single-winner public procurement system which does not explicitly consider quality has slashed the price paid for covered medicines. Trade, industrial and religious policies simultaneously increased production costs, pressuring profit margins for manufacturers and distributors. They reacted by cutting costs (potentially threatening quality) or by market withdrawal (leading to shortages which provide a market for falsifiers). Shortages and physician-promoted irrational demand push patients to buy medicines in unregulated channels, increasing exposure to falsified medicines.

**Conclusion:**

Market factors, including political pressure to reduce medicine prices and healthcare provider incentives, can drive markets for substandard and falsified medicines. To protect progress towards UHC, policy-makers must consider the potential impact on medicine quality when formulating rules governing health financing, procurement, taxation and industry.

**Key questions**

**What is already known?**

- The WHO suggests that substandard and falsified medicines are found where access to affordable, quality-assured medicines; technical capacity; and good governance are all limited. Universal health coverage (UHC) aims to expand medicine access, but little is known about its impact on medicine quality.

**What are the new findings?**

- In Indonesia where a rapid but under-financed scale-up of national health insurance took place, new procurement and reimbursement policies coupled with nationalist economic policies have squeezed profits for pharmaceutical manufacturers/distributors and healthcare providers.
- To protect profitability, some pharmaceutical manufacturers and distributors cut costs (increasing the risk of substandard production or degradation), or withdraw from the market (leading to shortages which provide a market opportunity for falsification).
- Some healthcare providers also maximise profits by creating irrational demand for premium products; this can push patients into the unregulated supply chain where falsified products are more common.

**What do the new findings imply?**

- Market factors are influential determinants of medicine quality. To ensure access to quality-assured medicines while aiming for UHC, policy-makers must take this into account, avoiding formulating policies around health financing, procurement, taxation and industry that incentivise the production or sale of substandard and falsified medicines.

## Introduction

For several decades, the global health community has worked to increase access to medicines, with efforts centred on affordability, especially in low- and middle-income countries (LMICs). Recent reports indicate that many of the medicines circulating in LMICs are substandard or falsified.^1 2^ These medicines often harm patients; they also waste money, contribute to antimicrobial resistance and undermine confidence in health systems.^3 4^

In 2017, the WHO analysed the first 1500 cases reported to the WHO Global Surveillance and Monitoring System for substandard and falsified medical products. WHO suggested that substandard and falsified medicines exist where constrained access to affordable, safe and effective medical products intersect with limited technical capacity to ensure good manufacturing practice, and/or corruption and poor governance in health and judicial systems.^5^ The analysis did not differentiate between drivers of substandard medicines (which are made by registered manufacturers, but which do not meet quality standards set out in their marketing authorisations, because they are poorly made or have degraded) and drivers of falsified medicines (which are illegally made, or repackaged so that they misrepresent the product’s contents, identity or source).

In every country, access to medicine, technical capacity and governance are substantially shaped by wider political and economic factors. However, the relationship between those contextual factors and medicine quality outcomes is not well understood. A clearer understanding of this relationship may help identify policies that create vulnerabilities, and suggest actions to reduce the risk that patients are exposed to substandard or falsified medicines.

In order to contribute to this understanding, we investigated the political and economic factors that drive production and trade of poor-quality medicines in four middle-income countries.^6^ Here we report in detail on a case study in Indonesia. Indonesia, the world’s fourth most populous country, was chosen for two reasons. First, it embarked in 2014 on an ambitious programme to achieve universal health coverage (UHC) by providing national health insurance coverage to its 280 million citizens by 2019 through a programme known as *Jaminan Kesehatan Nasional,* or JKN.^7–9^ Health financing and pharmaceutical procurement were extensively reformed, with potential consequences for medicine quality.

Second, Indonesia in 2016 experienced a widely-publicised case of vaccine falsification, which resulted in approximately 1500 children being injected with fake products.^10 11^ We reasoned that a careful examination of this case would provide a clear ‘micro-level’ entry point for investigation of the more ‘macro-level’ political and economic factors shaping medicine quality during the period of rapid scale-up of UHC.

## Methods

### Study set-up and participants

For this in-depth case study, we combined document analysis and interviews with purposively selected key participants. We analysed peer-reviewed publications, news reports, government regulations and presentations, court records and technical reports from development institutions. To maximise the potential utility of the study, we solicited input around policy interests and ethics from the National Medicines Regulatory Authority (NMRA) at the study planning stage.

Full details of our methods reported following COREQ (COnsolidated criteria for REporting Qualitative research) criteria, together with topic guides and coding tree, can be found at https://doi.org/10.7910/DVN/CVPSBB. We purposively selected heterogeneous key participants with in-depth knowledge of the 2016 falsified vaccine case or of: the pharmaceutical business in Indonesia; prescribing practices; the medicine regulatory environment; pharmaceutical quality assurance; the implementation of JKN at the national or subnational level.

### Data collection

Using a topic list, the lead researcher (AH) and/or EP conducted interviews in person or by phone from December 2017 to May 2018. We provided the participants with detailed information about the study purpose and confidentiality procedures before the interview and obtained written or recorded consent from all participants. Interviews were conducted in Indonesian or English and lasted between 45 and 90 min. Nine participants declined recording, but gave written consent for note-taking.

### Data analysis

Data were transcribed and coded using NVivo 12 software^12^ by the first author (AH); another team member (EP) coded a subset of interviews in parallel—differences in coding were discussed until a shared understanding was reached. Using constant comparative method of analysis,^13^ we combined themes from the coded interviews and document analysis to develop a rich and coherent narrative of the vaccine and JKN case. Following a grounded theory approach, we then identified and described the political and economic drivers of poor-quality medicine.^14^

We presented preliminary results at feedback meetings with the medicine regulator, development agencies and an informal study advisory group. We incorporated their feedback to enrich our analysis and policy recommendations.

### Patient and public involvement

We sought the views of patients, and parents of vaccinated children, as participants during the study, but they were not specifically involved in the design, conduct, reporting or dissemination of what is largely a policy-focussed investigation.

## Results

### Characteristics of study participants

We interviewed 31 (n=31) key participants from different professional backgrounds, detailed in table 1.

**Table 1:** Study participants, by professional role

Roles/professions	Number of participants
Healthcare providers	8
National government/Ministry of Health	3
Subnational government	1
Medicine regulator	2
Technical agencies	2
Manufacturers/pharma industry group	4
Distributors	2
National insurer	1
Academic	2
Patient, media, civil society	6

We report first on the details of the vaccine case, then on the broader landscape affected by the scale-up of national health insurance.

### The falsified vaccine case: incentives at the micro-level

In June 2016, Indonesian police announced that they had arrested seven people suspected of making and selling fake vaccines. Shortly thereafter, the NMRA issued a statement saying that suspected falsified vaccines have been found in 37 private sector health facilities across nine provinces of Indonesia.^15^ Police investigations later determined that falsifiers had refilled used imported vaccine vials, collected by an organised network of hospital cleaners.^10^

The Indonesian Ministry of Health (MOH) runs a well-functioning immunisation programme. Free vaccinations are provided in the public health system, and community health workers actively encourage participation. A domestic manufacturer makes all of the mandatory childhood vaccines; all are quality-assured through WHO prequalification.^16^ At the time of the falsified vaccine case in 2016, there was no reported shortage of quality-assured domestically produced vaccine in the public system.^17^

Private healthcare providers may also charge parents to immunise their children using non-programme vaccines. These tend to be imported products. Market data from 2016 record 10 branded or generic vaccines sold by the single domestic producer of vaccines, and 37 imported vaccines. Among paediatric vaccines, list prices for domestic products ranged between US$0.2 per dose for polio to US$10 per dose for a pentavalent vaccine. For imported vaccines, they range from to US$4 per dose for a hepatitis B vaccine to US$48 per dose for a hexavalent product.^18^

In a press conference, the NMRA said it suspected falsification of 12 vaccines registered to the domestic manufacturer and two multinational manufacturers. However, an official statement from the regulator later confirmed that all domestically produced vaccines were safe.^16 19^

Disruptions in manufacturing at the two named multinational manufacturers from late 2015 to early 2016 caused a shortage of several imported vaccines for children.^10^ Yet demand for these products persisted. One participant, an academic who studies medicine regulation, underlined the cultural tendency of Indonesian patients to follow advice from physicians without asking for more information; physicians in the private sector actively promoted imported vaccines as having fewer side effects than domestic equivalents. This, and the high price charged, contribute to patient perceptions that imported vaccines are better than alternatives available at no cost under government programmes.

“In my opinion, if a vaccine is more expensive then it’s automatically better quality, and that’s that.”

Mother of an infant

#### The profit motive

The introduction of national health insurance in 2014 capped the amount paid to health facilities for insured patients across a wide range of services. This reduced income in particular for private hospitals and physicians, which had previously commonly set tariffs as high as the market would bear.^9 20–22^ Some made up for lost income by promoting demand for medicines and procedures not covered by the scheme.

Q: If I can get something for IDR [Indonesian rupiah] 300, why would I pay 48 000?

A: The consumer, they don’t have the right to choose. Because everything is decided by the physician… And in this country, physicians offer medicines …mostly on a conflict of interest. The more they prescribe, the more they get the opportunity to travel, attend conferences, etc.

Pharmacologist

Regulation of procurement in the private sector is sometimes lax.^23^ Participants explained that some physicians in private hospitals bypassed hospital procurement to source their own medicines, then charged above list prices. A consumer advocate said parents reported paying for vaccines directly to the physician’s bank account instead of paying the hospital.

When shortages of imported vaccines occurred in late 2015, facilities found it harder to procure the products from appointed distributors. Shortly thereafter, freelance sales agents supplied by falsifiers began approaching surgeries in private hospitals directly, offering imported vaccines at low prices. Since some physicians were buying on their own account, cheaper vaccines translated into larger profits.

“Those physicians should have been suspicious from the start because of the cheaper prices [of falsified vaccines] …but instead they just praised the Lord for this, and thought of the profits.”

Former medicine regulator

These private sales channels result in a lack of accountability on the part of hospitals.

“One of the directors [in hospital X] said that the physicians purchased the vaccines without the hospital’s knowledge …from March to June 2016… Meaning, for 3 months, the hospitals did not know that there were any purchases of vaccines [outside their authorised system]. Every [falsified] vaccine purchase was the sole responsibility of the respective physician.”

Civil society activist advocating falsified vaccine case

#### Unclear oversight, limited punishment

Participants explained that at the time of the falsified vaccine case, regulatory oversight of the medicines supply chain was unclear, hampering detection and effective investigation. The regulator was responsible for the quality of products made by legitimate manufacturers, but had limited power to investigate falsified products, which were a matter for the police. Regulators could not easily access health facilities for post-market surveillance, since these were under the authority of the MOH.^24–26^

“Regulation-wise, at that time, we were not allowed to inspect pharmaceutical services in hospitals. The parliament pushed us to do more to investigate [the falsified vaccines]. But … we could not get into those hospitals’ systems.”

Former medicine regulator

Criminals were not dissuaded by the penalties for medicine falsification. The health law of 2009 sets the maximum penalty at IDR 1.5 billion (currently US$101 000).^23^ However, in 2013 falsifiers of an imported vaccine were fined just IDR 1 million (US$96).^27^ This compares with profits of up to IDR 25 million a week made by individual members of the vaccine falsification ring in 2016, according to court documents and police investigators.

The 2016 scandal was the result of irrational demand from patients, fuelled by profit-maximising physicians who took advantage of poor governance in the private health sector to buy vaccines from unregistered suppliers. Gaps in regulatory structures and historically low penalties allowed criminals to exploit these weaknesses. The case was a watershed, resulting in fines of up to IDR 1 billion (US$ 75 000) and prison sentences of 6 to 10 years for 13 people. (For documentation of all of the court proceedings (in Indonesian), see: https://doi.org/10.7910/DVN/CVPSBB).

While regulatory structures have since been greatly strengthened, many other factors that threaten medicine quality persist. We therefore turned our attention to the wider health system, as it strives to deliver UHC.

#### The broader health system: incentives at the macro-level

In 2014, Indonesia’s newly-elected president promised to provide national health insurance coverage to all citizens by 2019.^28^ The system provided for full coverage and free medication for almost all conditions, for a monthly contribution ranging from IDR 25 500 to IDR 80 000 per person (US$2.15 to US$6.74).^9^ Contributions for the poor are paid by the state. The system was in deficit from its first year, despite new procurement regulations designed to control medicine costs.^7 29^

#### Falling revenues for pharmaceutical manufacturers and distributors

**Figure 1:**
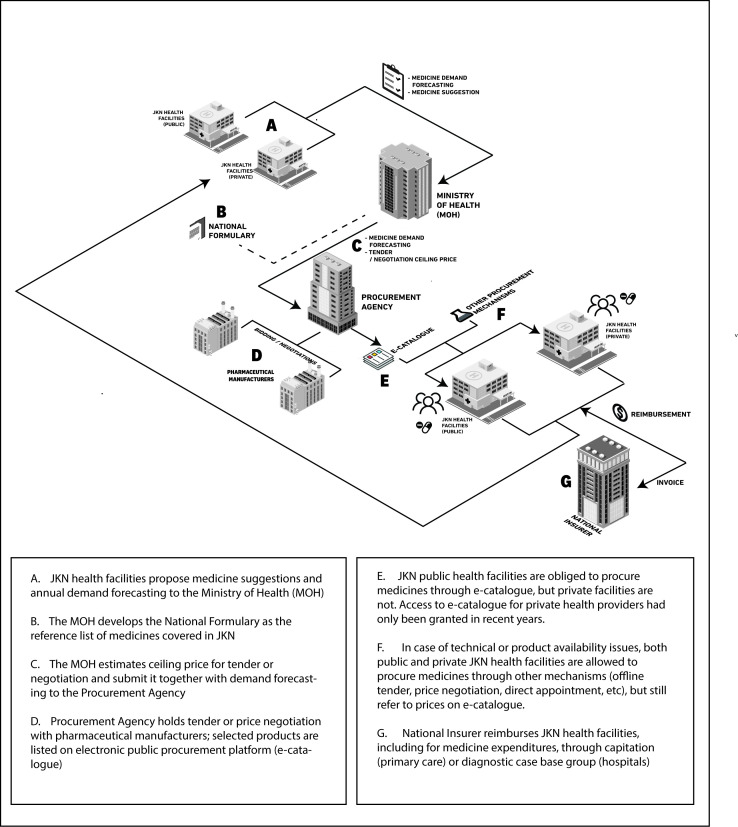
Summary steps in the procurement of medicines in the JKN system. JKN, Jaminan Kesehatan Nasional.

The new procurement system consolidated the market for all JKN patients, allowing manufacturers to bid to supply medicines for 1 year (2 years since 2018). The MOH forecasts demand and sets a ceiling price, and manufacturers bid (or, for innovator medicines, negotiate) to supply specific provinces. Figure 1 shows the simplified version of medicine flow within public procurement system in JKN.^21 22 30–33^ For all but a handful of medicines, there is only one winner per medicine per province, although companies commonly win contracts for several provinces, or the whole country. Bidders must hold a valid market authorisation, and must undertake to supply up to the volume forecast. With those conditions met, contracts are awarded on price alone.

The Indonesian pharmaceutical market is dominated by domestic manufacturers (88% of 206 registered companies).^34^ The expansion of JKN has increased the use of healthcare, escalating the overall volume of medicines sold. However, prices have plummeted, so in value terms the picture is less clear.^21 34 35^ By 2016, prices had fallen for almost 80% of 539 medicines publicly procured under JKN, many steeply.^30^ For example, amlodipine prices were slashed by 80% by 2018; simvastatin fell by 60%.^36^ In 2020, the public procurement price for amoxicillin 250 mg caplets, at IDR 195, was 55% less than New Zealand’s, the regional benchmark for competitive public pricing among countries with UHC.^37 38^

"In 2014, the MOH predicted that with JKN the pharma market would grow by 40%, but in fact growth in value terms actually dropped, to the mid-single digits. Volume is increasing, but market value not so much.”

Pharmaceutical industry representative

While pharmaceutical companies must promise to deliver the contracted volumes, the government does not undertake to buy those volumes. According to a former government official, the MOH does not use volume data from the procurement agency or claims data from the insurer in demand forecasting, instead compiling data only from health facility reports. Actual procurement often differs substantially from forecasts.^30^ In 2017 the government bought 30% of the forecast amount of paracetamol, while for iron folate, purchases exceeded forecasts by over a quarter.^36^

Manufacturers complain that inaccurate demand planning leaves them with unwanted stock, eating in to revenues. Some bidders now take their commitment to meet forecast volumes less seriously than in early years.

“We refer to the [government] demand planning… …but I also look at the market research data. To forecast production, you can’t look just at the demand planning. If it’s right, great. But if it is not? Where are you going to sell all those drugs?”

Pharmaceutical manufacturer

Delayed reimbursement from the national insurer to hospitals cascades through late payment to distributors, further constraining manufacturers’ cash-flow.^39^ In August 2018, the domestic pharmaceutical manufacturers’ association sent a complaint letter to the MOH demanding an immediate settlement from the insurer to hospitals, and later to distributors and manufacturers.^40^ The association reported IDR 3.5 trillion (US$246 million) worth of debts outstanding for over 1 year to local pharmaceutical manufacturers. The cash flow constraints are aggravated by an average 24-month lag in repayment of Value Added Tax.^40 41^

#### Rising costs

While prices fell, several policies from outside the health sector potentially increased production costs for the pharmaceutical industry. Wanting to boost the national economy, the government established local content requirements for publicly procured goods including pharmaceuticals.^32 42^ Since Indonesian manufacturers import 95% of their active pharmaceutical ingredient (API), this caused consternation.^21 34^ The implementing regulations for pharmaceutical companies, eventually published in 2020, in fact, provides considerable leeway in the way ‘domestic content’ is calculated.^43^ In interviews, however, both pharmaceutical executives and a former medicine regulator said that meeting the requirements would increase costs.

In addition, the Indonesian parliament enacted a populist-driven Halal Product Law that requires all medicines to be halal certified by 2019.^34 44^ Participants found this similarly worrying. While manufacturers with existing halal certification will benefit in the short run, those without could incur significant costs if the law is enforced.^21^

“The Minister of Health told parliament—and she’s quite right—should this [Halal Law] be enacted, medicine prices will rise. …It is difficult to find halal-certified APIs. Medicine prices could rise by one and a half to two times.… It is possible that [exporters] will boycott us. Europe has said should this regulation be enforced, they will withdraw themselves from the Indonesian market.”

Former medicine regulator

#### Net effect 1: pressure on profit margins erodes quality

Table 2 summarises the main factors decreasing revenues and increasing production costs in the Indonesian pharmaceutical market.

**Table 2:** Factors pressuring profit margins

Factors that decrease revenue	Factors that increase costs
Downward price pressures on medicine Inability to secure sales due to inaccurate demand planning on public procurement channel Constrained cash-flow	Local content policy—including for active pharmaceutical ingredients Mandatory halal certifications for medicines Local currency (IDR) devaluation

IDR, Indonesian rupiah.

Participants said that pharmaceutical companies respond to the erosion of profit margins in several ways that may compromise medicine quality. The most dangerous is cutting production costs, especially those related to raw materials and packaging.

Q: Is there an effect, where, because of the low offering prices, the components of medicines are compromised?

A: Yes, definitely. Starting with the raw materials. That’s the first thing, manufacturers are going to look for the very cheapest API, they’re going to look for a cheaper supplier. Next is the way they make the medicines available. For example, they might have started with blister packs, but they’ll change those to strips, something cheaper. Basically, they’re looking for ways to make more profit.

Pharmaceutical manufacturer

The risk for quality is observed by healthcare providers:

“Sometimes we receive medicines [for JKN patients] with poor packaging. Look at this strip packaging: it’s supposed to be sealed. But some medicines come with an already opened strip… so air gets in…. Medicine like this is not fit for consumption”

Apothecary assistant at primary care services

To cut costs, some market authorisation holders outsource production to smaller companies. This is legal, as long as it is stated on the market authorisation, and the contractor is certified for Good Manufacturing Practice (GMP).^45^ However, this is not always the case. One former regulator said that some smaller manufacturers continued to operate even after the withdrawal of their GMP certificates.

“How come these factories were still running? …It turns out they got production orders from bigger manufacturers…The dangerous thing was these bigger manufacturers did not use their own APIs, they just ordered [finished products] directly from the smaller one…So, it was like ordering food in a restaurant. The bigger manufacturers only see the end product. They did not want to know what kind of APIs were used.”

Former medicine regulator

Participants said distributors also cut costs in response to tight distribution margins, causing localised shortages and increasing the risk of degradation, for example, reducing investment in temperature-monitored storage for injections.

#### Net effect 2: flight from the market creates shortages, with attendant risks for quality

When ceiling prices (calculated by the MOH using an unpublished formula) fall below a threshold covering the cost of quality-assured production, distribution and fair profit, some companies anticipate reduced profit margins and refrain from bidding in public auctions, according to an industry executive. In 2018, auctions failed nationally for 253/1001 products, though 133 of these were later successfully rebid. A further 155 auctions failed for at least some provinces.^46^ At least one multinational manufacturer of quality-assured generic medicines has ceased its commercial operations in Indonesia, and many domestic companies report struggling.^21 40 47^

Reimbursement for JKN patients is mostly fixed by capitation or diagnostic group, and must include the cost of medicine whether or not it is available through the public procurement platform.^21^ Failed auctions thus leave health facilities to procure low-cost alternatives through other mechanisms.^30^ Some suggest quality may suffer.

If JKN medicines are out-of-stock, [our hospital procurement staff] must search for the same medicines from different manufacturers… and they look for the cheapest medicines. We receive other products from other manufacturers… …Previous products were packaged in a blister, a better packaging, but those new ones have strip packaging, which has lower quality.”

Apothecary assistant at hospital

Participants also reported cases in which the search for cheaper medicines disincentivised due diligence, increasing opportunities for dumping of degraded, expired or repackaged products.

Shortage of publicly-procured medicines facilitates the already common practice of encouraging patients to pay for medicines out of pocket.^20^ This sometimes drives patients to shop in more affordable but unregulated outlets, increasing the risk of exposure to falsified medicines.

“The physician told us to look for the medicines outside the hospital since they have an insulin shortage. We received the [diabetes] medicines from the hospital after an inpatient care. But, for outpatient treatment, they asked us to buy the medicines outside the hospital… …So, many patients try to look for insulin in the [informal] medicine market.

Chronic disease patient

Localised shortages are common even when auctions have not failed. The procurement system groups provinces into six regional blocks meant to reflect cost of distribution, allowing for a fixed increment for each block. The highest (applying to the remotest areas) is set at 20% above the lowest, regardless of product value. For low-value items, this small increment does not cover the cost of distribution across the 17 508-island nation, discouraging distributors from shipping to remote areas.

“As a brand owner, I make a marketing forecast… What is most important is that we still make a profit. Since I know the Cost of Goods Sold will be higher because the distribution chain to [the easternmost province] Papua is longer with higher costs, our margins will be reduced. So, I allocate more drugs to nearby areas, which are more reachable.”

Pharmaceutical manufacturer

Table 3 summarises profit protection by manufacturers, distributors and healthcare providers in JKN and the risks for medicine quality.

**Table 3:** Profit protection by actors and risks for medicine quality

Actors	Profit protection	Risks for medicine quality
Manufacturers or distributors	Cut production costs: Use cheaper active pharmaceutical ingredients	Substandard products at point of manufacture
	Cut production costs: Use cheaper packaging materials	Downgraded packaging, prone to degradation
	Cut production costs: Unregistered contract manufacturing to smaller factories without valid Good Manufacturing Certificate	Substandard products at point of manufacture
	Cut distribution costs: Limit supply to remote areas	Localised shortage, creating market opportunity for falsification
	Cut distribution costs: Reduce investment in temperature-monitored storage for injections	Downgraded storage, prone to product degradation
	Withdraw from market (including non-participation in public procurement)	Shortage, creating market opportunity for falsification
Healthcare providers	Prescribe premium and uncovered medicines	Irrational unmet demand of certain products or brands, creating market opportunity for falsification
	Select cheapest possible products especially during JKN medicines stockouts	Downgraded packaging, prone to degradation
	Limit medicine dispensed to patients	Push patients to unregulated supply chain, more vulnerable to poor-quality products especially falsified ones

JKN, Jaminan Kesehatan Nasional.

### Regulatory oversight

Indonesia’s NMRA, which also regulates food and cosmetics, runs quality control laboratories in 33 of Indonesia’s 34 provinces, employs over 3700 staff and in 2018 had a budget of more than IDR 2.17 trillion (currently around US$148 million).^48^ The agency issues market authorisations, works with the MOH to certify and licence pharmaceutical manufacturers and conducts regular inspections of factories, issuing sanctions as necessary. It also performs annual post-market surveillance based on public health risk. Until the vaccine case, the NMRA’s ability to prevent, detect and respond to threats of falsification were constrained by complex governance arrangements which, for example, restricted oversight of hospitals and public health facilities.^24–26^ After that case, structures were changed and new regulations issued, and progress has been made in streamlining procedures for post-market surveillance.^49–52^

The work of the regulators is not, however, taken fully into consideration by other agencies, especially those involved with procurement. With the exception of a valid market authorisation, which is issued for 5 years, quality assurance is not explicitly considered in public procurement criteria.^32^ While the regulator specifically includes JKN medicines in its post-market surveillance, the national procurement agency does not, in following rounds of procurement, take into account regulatory infractions such as testing failures during post-market surveillance, or documented violations of good manufacturing practice.

To ensure continuity of supply, winners of public procurement tenders face penalties ranging from warning letters to disqualification from future tenders if they do not fulfil their contractual obligations.^53^ Implementation of the measures is, however, unclear,^21^ and sanctions for failure to deliver to remote areas are rare.

## Discussion

This study is the first to investigate political and economic drivers of poor-quality medicines in Indonesia. We looked in detail at a specific case of falsification, as well as at the broader landscape of risk in the context of the rapid scale-up of public health insurance. Our results confirm a previous global analysis by WHO, which indicates that limited access to affordable, quality-assured medicines, limited technical capacity for production and poor governance contribute to the risk that substandard and falsified medicines will reach patients.^5^

Our study also identified drivers of falsified and substandard medicine that are not clearly articulated in the WHO’s analysis. These relate principally to markets, and other incentive structures that drive the behaviour of companies, institutions and individuals involved in the production, supply and consumption of medicines.

In the vaccine falsification case, free, quality-assured vaccines were universally available. There was no ‘unmet need’ for imported vaccines. There was, however, unmet *demand*, created largely by some physicians who profit by promoting expensive brands. This reinforced patient perceptions that equate branding and expense with quality—something that researchers have identified as creating irrational demand for costlier medicines in other markets, including China.^54 55^ Recently, a registered distributor in Indonesia took advantage of this dynamic, repackaging JKN generics as fake branded products.^56^ Previous research in Indonesia has identified this profit-maximising as driven in part by the reduction in physicians’ earnings that accompanied the scale-up of national health insurance.^22^

Some physicians were also willing to buy ‘premium’ products from freelance salespeople at a discount. While there was indeed a shortage of imported products on the national market, these healthcare providers stepped out of the regulated supply chain largely to increase their profits. As predicted in WHO’s framework, this unethical behaviour provided an entry point for falsified medicines. Poor governance within hospitals and institutional curbs on regulatory oversight allowed falsification to go virtually unchallenged for several years. To tackle policy issues around unmet demand and incentives, we recommend further studies to investigate the externalised impacts of physician and hospital compensation on patient well-being, including exposure to poor-quality medicines.

One other major finding significantly expands the WHO framework: profit protection by pharmaceutical companies and distributors can incentivise the production of substandard medicines, as well as creating shortages which may be filled by falsifiers. Companies acted to protect profits largely in response to new procurement rules introduced to reduce medicine prices in the public sector, helping the government to deliver on its political promise to achieve universal health coverage. We found that prices that manufacturers view as excessively low incentivised cost-cutting activities, compromising quality assurance and good distribution practice; this corroborates findings recently reported by Wasir and colleagues.^22^

We also found that companies will simply pull out of auctions, market sectors or entire markets that they judge to be unprofitable, triggering shortages. Constrained access to affordable medicines is widely recognised as creating opportunities for falsifiers. However, shortages are most often attributed to spikes in demand caused by disease outbreaks, combined with regulatory and logistic hurdles, shortage of raw material and poor inventory management.^5 54 57^ The solutions proposed often include technical measures such as establishing an information system facilitating stock monitoring and communication between diverse actors.^54 58^ While we agree that improved demand forecasting would reduce shortages, we underline the important role that corporate incentives play as a driver of shortages. A similar study in Romania found that rapid changes in procurement and pricing policy led to the withdrawal of around 2000 of 6200 registered products.^6^ Multinational producers are particularly likely to withdraw or withhold products because their calculus includes the possible reduction of profits in higher-margin markets, through parallel exports or price benchmarking.^59^

The imperative to reduce medicine prices in Indonesia, the world’s fourth most populous nation, became a focus of political attention, growing along with JKN’s well-publicised deficit.^60^ Similar dynamics are observed in a number of middle- and lower-income countries as they try to increase public health provision.^54 61–64^ We find that if these efforts eliminate what companies consider a reasonable profit, medicine quality is likely to suffer.

More transparency from pharmaceutical companies about actual manufacturing, development or marketing costs, and from governments and insurers about prices paid for medicines, would greatly facilitate more productive discussion about what constitutes a fair profit.^65–68^ It is clear, however, that price calculations must include the cost of complying with good manufacturing and distribution practice, and other aspects of quality assurance. In Indonesia, we strongly encourage the adoption of explicit quality assurance criteria in the procurement of medicines (a possibility that is currently under discussion).^69^

Many studies of poor-quality medicines in LMICs conclude with a call to strengthen the national medicine regulator.^70–72^ While the NMRA encounters gaps in infrastructure and technical capacity across different provinces in Indonesia, the agency has been greatly strengthened since the vaccine case.^73 74^ Our study highlights the role of market factors, industrial policy and other politically-driven policies in increasing the risk of substandard production, degradation and falsification of medicines. In Indonesia, as in most other countries where the same factors are likely to be at play, the medicine regulator has no authority in these areas. While acting as a keystone for the enforcement of quality assurance, medicine regulators must thus coordinate closely with other institutions to reduce the incentives that drive falsification and substandard production.^75^ Political commitment to achieving UHC may provide the power to convene actors across sectors, and to balance competing objectives by taking a system-wide perspective.^58 76^ We believe that medicine quality is a joint responsibility, which requires a strong coordination and clear division of roles and tasks between different institutions.

Our qualitative study allowed for the detailed investigation of factors shaping the behaviours of medicine producers, distributors, providers, consumers and regulators. Interviews, conducted at both national and subnational levels, were limited in number, and a few participants were rather normative. However, we were able to triangulate information between participants, including about unethical practices and the limitations of governance structures. This may have been because we took the much-publicised case of vaccine falsification as a starting point. Large variation of study participants with quite small numbers on each group curtailed a consensus within each group, but we identified a general thematic saturation between different groups. We received feedback from the national regulator and others during our analysis, enriching our understanding, and were also able to validate some statements (for example, about pricing and shortages) with quantitative data. Health service provision in Indonesia is highly decentralised, so some of the findings relating to patient-level risk may not be representative. However, the national insurance system, including its reimbursement and procurement mechanisms, are centralised, meaning that there will be less variation in their effects nationwide.

## Conclusion

Our study shows that in Indonesia, market factors, including political pressure to reduce medicine prices to help achieve UHC and healthcare provider incentives, can be influential determinants of medicine quality. The risk of substandard production and degradation rises when revenues earned by legitimate manufacturers do not adequately cover the cost of quality-assured production and fair profit. The risk of falsification rises as shortages become more common following market withdrawal; they are greatly increased by profit-maximising healthcare providers who promote irrational demand and neglect due diligence. Taking these factors into account when formulating policies around medicine procurement, reimbursement, taxation and industry would complement existing product regulation measures, help further secure access to quality-assured medicines for Indonesian patients as the country works towards achieving UHC.

## Data Availability

Data are available upon request. The interviews that underlie in this study discuss at times unethical behaviour. During our informed consent procedure, we assured participants of anonymity. We are unable to comply with that commitment if we make the interviews recordings available. We believe there is an unacceptable high risk of disclosure in sharing full transcripts, and do not have the resources to redact the interviews fully. However, we do provide our detailed coding structure (https://doi.org/10.7910/DVN/CVPSBB). Researchers are welcome to request specific coding queries by contacting the corresponding author. We will run the queries as requested, redact the results only to the extent necessary to ensure anonymity and pass the results on to fellow researchers. We also provide a downloadable bibliography of all references reviewed during this research, and a collection of full documentation of all court records from the falsified vaccine case.
